# Does the current crisis mark the end of the EU’s austerity era? Competing political projects in European fiscal governance

**DOI:** 10.1057/s41295-023-00346-4

**Published:** 2023-04-28

**Authors:** Joscha Abels

**Affiliations:** Institute of Political Science, University of Tübingen, Melanchthonstraße 36, 72074 Tübingen, Germany

**Keywords:** Austerity, Economic and Monetary Union, Euro crisis, European integration, Fiscal policy, Inflation

## Abstract

Following the pandemic, the EU has responded to the threat of a euro crisis flare-up by deactivating its fiscal framework and establishing the Recovery and Resilience Facility, drawing on joint European bonds to finance national investments. This paper seeks to explain these modifications to fiscal governance and asks whether they are an indication of European austerity making way for an alternative fiscal paradigm. Based on a neo-Gramscian approach, it discusses the policies as parts of competing political projects that are promoted or hindered by certain framework conditions. The paper undertakes a structured comparison of these framework conditions during the euro crisis and the current crisis. It finds that geoeconomic competition increases the demand for a more active fiscal policy, while political preferences and structural relations remained remarkably stable. As the current crisis is marked by high inflation, economic conditions are adverse to a fiscally expansive agenda. The findings do not suggest a lasting reorientation of European fiscal governance. Instead, the measures taken during the pandemic are interpreted as expressions of ‘passive revolution’ in which the EMU adapts elements of a fiscal integrative agenda to provide necessary fixes to its economic order while keeping its underlying fiscally restrictive features intact.

## Introduction

The EU’s history is a history of crises and the leaps of integration they triggered. This has yet again been underlined by the economic fallout of the pandemic and the measures the EU took to counter it. In March 2020, the Commission triggered the Stability and Growth Pact’s (SGP) ‘general escape clause’ for a first time. The clause classifies COVID-19 as an extraordinary event, allowing the member states to deviate from their debt and budget targets. It will remain active at least until the end of 2023. Against the initial resistance of some northern countries, the EU has also agreed on the Recovery and Resilience Facility (RRF). It consists of grants and loans totaling €750 billion to provide a stimulus to national economic recovery and structural transition, financed via the issuance of joint EU bonds. Remarkably, the member states broke with two constants of euro crisis management: rigorous fiscal consolidation and keeping check books separate.

These measures provided a tailwind to initiatives challenging European austerity which had characterized crisis management in the eurozone thus far. French president Emmanuel Macron and Italian prime minister Mario Draghi demanded a “new fiscal framework” that should involve further joint European investments “given that public spending of this sort actually contributes to debt sustainability over the long run” (Draghi and Macron 2021). Most remarkably, a joint paper by the Dutch and the Spanish finance ministries calls for a reformed fiscal framework that would “increase high quality public investments” (Government of the Netherlands 2022). Back in euro crisis days, the Netherlands and Spain represented opposite camps, with Dutch finance minister Dijsselbloem as Eurogroup president pushing a hard consolidation line (Abels 2019). The core institutions of European economic governance have signaled their willingness to discuss reforms. The European Commission has initiated a consultation process on the Economic and Monetary Union’s (EMU) future form (COM (2021) 662 final). Leading economists of the European Central Bank (ECB) have proposed not a fundamental reform but a deceleration of fiscal consolidation to allow for more counter-cyclical spending (Haroutunian et al. 2022). Even senior economists of the European Stability Mechanism (ESM) have suggested to raise the SGP’s debt ceiling (Francová et al. 2021). However, the intergovernmental discussion of substantial institutional reform has recently died down, with the EU’s finance ministers again calling for “debt sustainability” and seeking to limit fiscal efforts to securing Europe’s energy supply (Eurogroup 2022). Germany has also rebuffed moves for common European fiscal instruments in response to the energy crisis (Chazan and Fleming 2022).

Academic debates are centered around the quality and transformative character of the observed shifts (Dullien 2022; Ferreiro and Serrano 2021; De la Porte and Jensen 2021), their implications for EU industrial and competition policy (Renda 2021; Schneider 2022; Wigger 2019), and potential further reforms (Heimberger 2020; Truger 2020). Proponents of a fiscal union have long underlined the need for a centralized European authority that might raise funds and support the EMU’s economies through fiscal instruments (Bordo et al. 2013). Some see the pandemic measures as a doorway for a more comprehensive reform of EU fiscal rules or at least some substantial adjustments. This paper seeks to explain the recent institutional developments from a neo-Gramscian perspective, asking whether they are an indication that the EU’s austerity-political paradigm is making way for an alternative fiscal project. In contrast to other accounts, it provides a more skeptical assessment. Its comparative-historical analysis demonstrates that the differences in economic and political conditions between the euro crisis and now explain the slowdown of the austerity project, but that they do not suggest a lasting reorientation of fiscal governance. The reforms following the pandemic should not be taken as an indication of the assertion of a political counter-project promoting a large-scale mobilization of EU resources and a persisting reprioritization of investments over consolidation. Rather, they form components of a ‘passive revolution’, a readjustment process that stabilizes the current framework by amending it with competing proposals and mitigating resistance.

In developing that argument, the paper proceeds as follows: The first section depicts the recent evolution of European economic governance and reviews academic works that have dealt with it. It highlights the quality of contributions but also their tendency to give greater importance to punctual developments than structural shifts. The second section then outlines a neo-Gramscian approach to the transformability of economic orders and their associated political projects. The third section characterizes European austerity as a political project and outlines its enabling conditions. The fourth section then undertakes an empirical comparison of the potentials of restructuring the EMU during the euro crisis and the current one. It proceeds along the lines of four criteria: economic conditions, geoeconomic competition, structural power relations, and legal-institutional arrangements. It finds that while geoeconomic context factors indeed favor fiscal expansion, political factors, most significantly the northern countries’ preferences and influence, have remained remarkably stable. What is more, institutional reforms during the euro crisis have further enshrined fiscal discipline and inflation is making it harder to promote the easing of this paradigm.

## Restructuring the EMU in times of pandemic and war

Some scholars view the crisis measures as a continuation of a general reorientation of the EMU that has begun already before the pandemic. Fiscal restriction had characterized European integration since its beginnings and was gradually intensified until industrial policy in its traditional form was marginalized (Bulfone 2022). Yet, employment and innovation in the EU are highly dependent on industry (Szczepański and Zachariadis 2019). As major European economies like Italy and Spain lost about a quarter of their industrial production in a wave of deindustrialization that accompanied the euro crisis, whereas the heavily industrialized economies proved much more resilient to crises, there was substantial pressure on the EU to promote re-industrialization (Pianta 2014).

The term ‘new European industrial policy’ describes an enhancement of industrial measures on the European level and an analogous recalibration of EU institutions. The underlying argument is that industrial policy measures, after an “industrial policy winter” (Renda 2021) in Europe, are having a comeback — and with them a more active fiscal stance. An adjustment of German preferences is considered central to the development. As Schneider (2022) finds, in light of geoeconomic competition from the US and East Asia the German ‘power bloc' has promoted strategic public investments and an easing of competition rules. Therefore, the market-based modus operandi, “long taken for granted in critical European integration studies, appears to be eroding, at least in regard to EU competition policy” (Schneider 2022: 14). Yet, Wigger (2019) warns that this process is not necessarily accompanied by a more fiscally active stance as it might be compensated by savings and internal devaluation elsewhere.

The economic measures in the wake of the COVID-19 crisis have shifted the debate even further toward the expectation that the EU fiscal policy is readjusting. In March 2020, the European Commission triggered the general escape clause of the SGP, temporarily allowing member states to deviate from their budget and debt targets. By classifying the pandemic as a ‘generalized crisis’, the clause is supposed to provide member states with fiscal leeway for public support measures. The member states also set up the RRF, allocating funds between them in relation to their individual recession and employment effects. Contingent on the approval by the European Council, the countries receive funds from the RRF for national investment plans. Northern countries like the Netherlands, Austria, and Denmark managed to shift a large part of the resources from grants to loans, effectively reducing the fund’s volume. Yet, they were unable to prevent debt mutualization. To capitalize the fund, the EU issues joint bonds via the European Commission, making use of its favorable market position. Ferreiro and Serrano (2021) view the RRF and the suspension of the escape clause as indicators that there is “a qualitative change in the EU’s approach to dealing with the negative consequences of economic shocks” (Ferreiro and Serrano 2021: 214). They find that the measures, in combination with the ECB’s Pandemic Emergency Purchase Programme (PEPP), were successful in cushioning the effects of the crisis. The authors go as far as speaking of a wider learning process in EU crisis management and predicting that a “change of [fiscal] rules is likely to be one of the economic corollaries of this health and economic crisis” (Ferreiro and Serrano 2021: 224). De la Porte and Jensen (2021), however, show that the RRF—as the most significant pandemic measure—was the result of one-off haggling rather than a paradigm shift. The northern states agreed to the fund only in exchange for side-payments like individual rebates. Their study also outlines the northern states insistence on the temporary character of the measures. Nevertheless, scholars are emphasizing the policy innovation which the RRF and its financing model represent. The EU issues joint bonds to finance the recovery fund and it opted to do the same for SURE, a job insurance instrument that supports national employment and short-time work schemes. Andor (2020: 141) thus views SURE as “a counter-cyclical fiscal capacity” and an initial step toward “a proper stabilisation role at the community level.” In light of these innovative—and highly successful—modes of raising European funds for the RRF and SURE, Da Costa Cabral (2021: 13) concludes that the EU is “mov[ing] aside from pure national borrowing models” and toward “a European hybrid solution.”

Overall, there is a number of insightful papers on the recent developments in European fiscal governance that shed light on the underlying political and institutional shifts. Their findings are sometimes contradictory, but they generally place an emphasis on the observation that the EMU is currently at a more or less pronounced turning point. This conclusion is often the product of rather ahistorical analytical and theoretical approaches that display some variant of recency bias: overvaluing contemporary developments compared to long-term path-dependencies. In the following section, I develop a critical political-economic perspective that focuses more strongly on structural and economic factors and, on this basis, inspires a more cautious assessment.

## The stability and transformability of political projects

I suggest viewing both the austerity-political restructuring after 2010 and the recent advances of fiscal expansion as parts of wider political projects. For that purpose, I draw on a neo-Gramscian framework that provides conceptual and analytical instruments to explain the recent shifts in fiscal governance and assess their implications. Within this frame, political measures and initiatives do not appear as isolated phenomena but are embedded in broader social structures. Political projects are understood as programmatic and practical materializations of specific interests and discourses (Bieling 2015). Their initiation requires opportunity structures that raise doubts about the current order and allow alternative ideas to gain a foothold. Thus, political projects can be understood as answers to severe problems or urgent crises. Ultimately, their proponents seek to establish the certain paradigms in a political space, that is to have the project reach ‘hegemonic’ status. In a neo-Gramscian understanding, hegemony does not merely consist of a material dimension, but also requires “intellectual and moral leadership” (Gill 2008: 91). It presupposes combining a certain economic order with a shared ideological framework that delivers a normative basis and ensures the consistency of problem perceptions and policy proposals. It also has to incorporate a variety of actors. Projects are carried by heterogeneous alliances of state apparatuses, political factions, fractions of capital, academics, and civil society groups. Their political interests are compatible due to their specific positions within the socio-economic order and a shared world view, whose problematizations and objectives the coalition tries to generalize through the project. In that process, they might aim to consolidate existing power relations and value systems, but also to readjust or even transform them.

As historical formations are characterized by inconsistencies, political alliances will promote alternative views and policies that challenge said formations. Again, crises play a major role as they lead to societal ruptures which social forces can exploit for counter-projects. The more systemic and disruptive the crisis, the more potential they represent for a comprehensive restructuring. However, there are ways in which political projects might overcome challenges without undergoing a substantial transformation. As a key characteristic, they are embedded within the legal-institutional framework of a political space—what Gill (1998) calls the ‘new constitutionalism’. European integration has inextricably woven principles of price stability, central bank independency, fiscal discipline, and freedom of capital and labor into the EU’s framework. The new constitutionalism creates a reliable basis for businesses and private investors as it shields them from the effects of changing political majorities. Even in scenarios of major disruptions due to crises and alternative discourses and policies gaining traction, they might fail to alter the persisting order due to the path-dependencies created by new constitutionalism.

There are, however, situations where resistance to the hegemonic order and its associated political projects becomes overwhelming and the dominant social forces are resorting to processes which neo-Gramscian works label as ‘passive revolution’. These can take the form of an “elite-engineered social and political reform” (Morton 2010: 318) that resolves a crisis situation in favor of the dominant forces without wider societal consensus. Alternatively, and more relevant to this paper, processes of passive revolution can co-opt and appropriate oppositional forces and the counter-projects they promote. They combine, to different proportions, progressive and restorative elements (Callinicos 2010). The former constitute measures and discourses that are extracted from rivaling projects and serve to absorb competing discourses and subordinate interests into the hegemonic project. Thus, the modifications to the constitutionalist framework rely on “a strategy of incorporating, and ideologically neutralizing, rival projects” (van Apeldoorn 2009: 22). There is a dialectic dynamic to this process. The progressive elements amend the current order and might entail genuine changes of the economic order. At the same time, they provide specific fixes that can have stabilizing effects on the superordinate structures. The underlying orientations and hierarchies are then conserved as alternative concepts are appropriated and put to use, with dividing effects on the alliances that support them.

## European austerity and its historical determinants

Several authors have suggested that European austerity after 2010 is best understood as a coherent political project, focusing their inquiry on its ideological underpinnings (Blyth 2015), its historical roots in the EMU architecture (Stützle 2014), its class-related aspects (Palley 2013), its political realization (Abels forthcoming), and its dependence on the shifts in the German economic model (Scharpf 2017). This paper does not seek to provide a comprehensive depiction of the historical development and the alliances carrying the project—the afore-mentioned works and others have made strong contributions on this. Instead, the following section undertakes a characterization of the project and sheds light on its underlying logic and drivers. A particular focus lies on the enabling conditions that made the project gain traction after 2010.

Rarely has an economic area been shaped by an economic paradigm as lastingly and effectively as the eurozone by austerity between 2010 and 2018. On both the national and the European levels, the restructuring of societies was characterized by remarkable consistency. In the course of the euro crisis, Greece, Cyprus, Ireland, Portugal, and Spain had to apply for multilateral bailouts in exchange for strict conditionality. Their adjustment programs featured a combination of structural reforms and privatizations and rested on take-it-or-leave-it offers to countries that were ultimately dependent on European funds. The crisis countries therefore had to readjust their economic models in line with the project’s conceptions—to different degrees, of course. In addition, the deteriorating situation in the crisis countries reconfirmed the member states that more comprehensive action was required. This paved the way for a ‘crisis constitutionalism’ (Bieling 2015): institutional reforms that reflected an intensification of new constitutionalism with specific attributes. While new constitutionalism is particularly pronounced in the area of monetary and fiscal policy, crisis constitutionalism substantially concerns economic policy. The tightened budget rules of the SGP keep national governments from implementing expansive policies, whereas the newly established European Semester and ESM provide instruments with which the EU can reign in national economic affairs. The setup of a comprehensive intervention apparatus added to the coercive component of the EMU’s institutional framework (Kreuder-Sonnen and Zangl 2015). Whereas the new constitutionalism could be considered a “by-product […] of market-liberal globalization” (Bieling 2015: 105), the nature of crisis constitutionalism is more disruptive.

When it comes to the wider objectives and the drivers of the project, an internal and an external dimension can be identified. Internally, the project shifted the adjustment pressure, emanating from a crisis that can generally be characterized as a structural crisis of the EMU (Aglietta 2012), toward the low- and middle-income households of the southern periphery. National crises which, to some degree, could all be retraced to dynamics of uneven development within the eurozone, were rebranded debt crises caused by profligate spending that exceeded productivity (Becker and Jäger 2012). Within this interpretative frame, the solution to the crisis was a balancing of budgets from the expenditure side, that is through budget cuts and a consolidation of debt levels that were considered ‘excessive’. This secured the support of financial businesses that, after having invested heavily in European periphery bonds, were receiving another, more indirect bailout (Blyth 2015). At the same time, the finance ministries of the northern countries politically organized the process as they too feared the eventuality of another banking crisis and sought to avoid a readjustment of their own economic models (Abels 2019).

Externally, the austerity project called for a harmonization of the EU’s economies under a common globalization strategy. This strategy, however, would be largely aligned with the model of the northern economies as it seeks to achieve export-led growth for the eurozone as a whole (Scharpf 2017). The targets set by both the national programs and the added institutions are biased toward a fiscally restrictive and export-oriented model. Through this sectoral shift and the preservation of northern export sectors’ price competitiveness, the project garnered the support of the world-market-oriented industries (Schneider 2022). Overall, the project of European austerity is rooted in the fiscal-restrictive and ordoliberal fundamentals of the EMU (Ryner 2015; Stützle 2014), but intensifies these principles in line with an austerity ideology that was dominant among political decision-makers (Abels forthcoming) and business elites (Palley 2013). While particularly pronounced in the crisis countries, it evidently has pan-European ambitions.

The austerity project’s persistence depends on a favorable constellation of contextual factors—and so does the progress of counter-initiatives. I identify four categories of factors that are contributing to or hindering the emergence and implementation of political projects. Respective factors played a more or less pronounced role in the realization of the European austerity project as well. The four categories also serve as a typology for the ensuing analysis. The list is derived from neo-Gramscian concepts and arguments. Albeit non-exhaustive, I deem it substantial enough to shed light on the circumstances defining the potentials of austerity and a fiscal counter-project after 2018.

First, *economic conditions*, most importantly macroeconomic developments, define the productive potential of political projects. In neo-Gramscian thought, it is above all periods of ‘organic’ crisis and persistent instability that lend themselves to pushes for social change. Even though they ultimately translate into different solutions, depending on prevailing ideologies and power balances, they determine the urgency and feasibility for action. The European austerity project seized first and foremost the recessionary developments in the European south and its debt crises to drive a restructuring of the eurozone as a whole.

Second, *geoeconomic competition* influences the prospects of a certain globalization strategy to which political projects contribute. As a transnational perspective, neo-Gramscianism links the political evolution of economic areas to their wider global productive context and the extant power relations. Shifts in global order, the rise of specific transnational issues, and insecurities about future economic relations all are enabling factors for political action. As was argued before, the austerity project seeks to harmonize the eurozone’s economies under a common export-oriented strategy. As a project that builds strongly on the opening of global markets and a rule-based economic order, it benefitted from the restoration of the market-liberal order after the global financial crisis and a period of relative geopolitical ease.

Third, as there are specific political alliances carrying a project, *structural power relations* within an economic area are defining the momentum they can reach. From a neo-Gramscian perspective, this concerns the structural power private businesses possess in global politics (Gill and Law 1989). Heuristically, it is worthwhile to investigate the influence of capital fractions and the specific demands they are articulating. In the eurozone, a highly integrated economic area in which national economies compete with each other, the member states and their associated state apparatuses bundle the interests of particularly influential national companies and sectors and represent them at the European level. The uneven development paths of the eurozone’s economies before 2010 have provided the northern countries with a strong financial and discursive leverage over European economic affairs which they used to drive austerity (Abels 2019).

Finally, taking into account the perpetuating effects of new constitutionalism, elements of the *legal-institutional framework* should be considered. The political paradigms enshrined in treaties and the rules and procedures of supranational and intergovernmental bodies predetermine what is politically feasible and which options are excluded (Gill 1998). The EMU implements economic policy through institutional arrangements rather than legislation as it provides the framework for action in a complex EU system. As was argued before, the austerity project built on preferences and rules embedded in the ordoliberal architecture of the EMU. Yet, an amendment and expansion of the framework were required to institutionally flank the project, which found its expression in crisis constitutionalism.

## Comparing the potentials of European fiscal projects

To explain the recent developments in EMU governance and assess the potentials of an alternative fiscal project, the remainder of the paper conducts a comparison of the current framework conditions of European economic governance and those prevailing during the euro crisis. The comparison is structured along the lines of the four afore-mentioned categories and is largely based on quantitative economic indicators, qualitative data from documents, as well as additional evidence extracted from reports and previous works. The analysis finds that the geoeconomic context has indeed changed in favor of a counter-project; yet, the overall control pattern renders mixed results. The inflationary dynamics and the constitutionalist framework even suggest a resurgence of austerity rather than its displacement. I conclude that the differences between crisis conditions explain the slowdown of the austerity project, but do not allow for a lasting reorientation—which finds its empirical expression in a lacking follow-up to the energy crisis and a gradual return to fiscal consolidation.

### Economic conditions

**Fig. 1:**
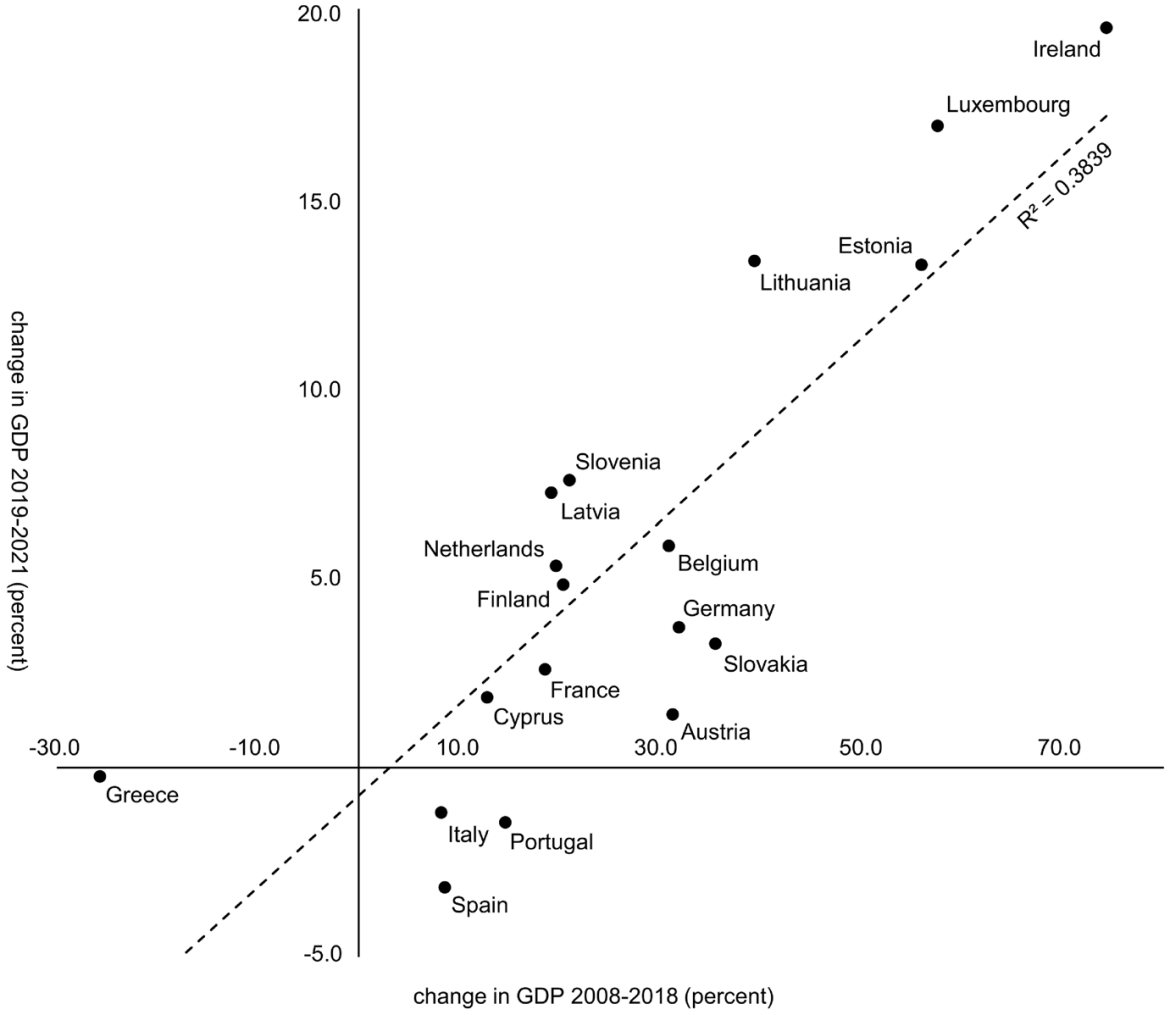
Change in GDP during euro crisis (2008–2018) and onset of current crisis (2019–2021) for euro area countries (source: Eurostat, own calculations)

The root causes of both the euro crisis and the current economic downturn are very different. The former constituted an extension of the global financial crisis, catalyzed by deindustrialization and a bust of debt-financed development in the southern periphery (Dullien 2022). The current crisis, on the other hand, has been triggered by a loss in production and disruption of value chains following the pandemic. Recently, it has been aggravated by shortages in the global supply of energy and goods caused by the Russian war in Ukraine. By now, the crisis simultaneously has the character of a supply- and demand-side crisis (Vernengo and Nabar-Bhaduri 2020). Still, the crises’ main symptoms are strikingly similar: a sharp increase in public debt—in 2010 because of the bank bailout, in 2022 due to spending mitigating the fallout of the pandemic; a widening spread in bond yields between member states, mostly to the disadvantage of the southern periphery; and output levels indicating recession. Despite some claiming otherwise (Da Costa Cabral 2021), the effects of the current crisis are again distributed unevenly across the EU. Southern countries like Italy, Spain and Greece went into the pandemic marked by high public debt levels and low aggregated growth over the last decade. As Fig. 1 shows, it is precisely these countries that are particularly affected by the current recession, clouding their economic outlook and raising public debt-to-GDP levels even further.

At the same time, pandemic measures have been particularly costly in the periphery (Gräbner et al. 2020). Considering a more restrictive monetary policy on the part of the ECB, debt burdens are set to again pose a serious stability risk for the EMU. The ECB is in the process of stepping away from its function as a de facto lender of last resort for the eurozone. The discontinuation of its asset purchase program PEPP has made refinancing conditions more insecure, contributing to a move of investors away from periphery bonds (De Grauwe 2022). Whereas the yields on long-term bonds in the periphery had fallen close to zero percent after 2018, leading to a relative convergence across the eurozone, toward the end of 2022 the spread between periphery bonds and northern bonds was again widening. At the end of December, interest rates for Greece and Italy were sitting at 4.5 percent, while Germany’s were just below 2.5 percent. Macroeconomic developments thus indicate an uneven fallout of the current crisis that resembles the economic asymmetries of the previous decade.

In contrast to these similarities, price developments differ significantly between both time periods. While the EU operated in a deflationary environment during the euro crisis—a consequence of falling wages and low aggregate demand—its concern now is a historic phase of inflation. In August 2022, the annual inflation had risen to 9.1 percent in the euro area. Although the development is driven by a hike in energy prices and costs of food rather than high demand or overheating production, the development has built enough pressure on the ECB to make it revisit its former course of monetary expansion. The central bank implemented an interest rate hike in July 2022, the first of several that saw its benchmark interest rate reach 3.0 percent in February 2023—to be followed by further hikes. As this makes investments more expensive for businesses, the ECB’s adjustment could deepen the looming recession. The end of monetary easing will reduce the knock-on effects of public spending and contribute to rising refinancing costs in the periphery as capital is looking for a safe haven (De Grauwe and Ji 2022). The fact that the ECB seeks to control a mostly supply induced inflation by curbing employment and investment poses a major obstacle to initiatives of fiscal expansion.

Overall, the external causes of recession and rising debts levels are more clearly visible this time around, which can be argued to have raised the initial acceptance for jointly financed counter-cyclical measures. However, the uneven impact of the crisis and, more importantly, the inflationary dynamics are providing the discursive foundation for attempts to blame said fiscal measures for undesirable developments, as visible in recent communicative strategies of Germany’s finance minister Lindner (2022). The ECB’s rate hikes in particular render fiscal measures less effective. All in all, economic conditions are currently as conducive to austerity-political action as they were at the beginning of the euro crisis. This time around, it is not so much the asymmetric debt levels as the inflationary conditions that serve as a discursive backing for fiscal restraint.

### Geoeconomic competition

While economic conditions do not favor a reorientation of the EMU, the geoeconomic context certainly does. During the euro crisis, the EU’s agenda rested on a deregulation of labor markets and a reduction of production costs for European companies. This was in line with the Lisbon strategy that sought to convert the eurozone into an export area marked by price competitiveness and high productivity. Processes of national ‘convergence’ were mainly defined in terms of “the structural functioning of the export-oriented northern economies” (Scharpf 2017: 290). This export-oriented globalization strategy relied strongly on conditions of free markets and a liberal global order. In recent years, several developments have drawn this market-liberal approach into question (Abels and Bieling 2022). Disruptions of international trade and manifestations of deglobalization render export-led growth more costly and risky. Global productive relations are marked by drops in demand and shortages of vital components for industrial production. After years of austerity, internal demand and consumption in the EU are insufficient to offset these losses. Hence, the EU is looking for ways to secure its supply with energy and components, while also struggling to stabilize its exports. Discursively, this has gone hand in hand with calls of influential intellectuals for ‘economic sovereignty’ (Leonard et al. 2019) that should make production in the EU more resilient and less dependent from the strategic decisions of external competitors.

This rethink has been fueled by shifts in global power relations, driven by the rapid growth of the Chinese economy and the assertion of state capitalist development models that combine a gradual integration in global markets with tight state control and protectionism (Nölke et al. 2015). The EU has benefitted substantially from trading with China: In 2021, China was the EU’s most important source of imports, while absorbing the third-largest share of European exports. However, the unequal market access granted by Beijing to European companies has created frictions. China has gained competitive advantages by using state financing to cut out European competitors or, in some cases, buy them up and absorb their technologies and know-how. The EU by now views China not just as a trade partner but also as an “economic competitor in pursuit of technological leadership, and a systemic rival” (JOIN(2019) 5 final). Relations with the US have also been strained as the member states have been the target of protectionist trade measures by the Trump administration (Scherrer 2022).

Although interests are realigning under the Biden administration, the EU is responding with an amendment—even partial replacement—of its market-liberal approach. Under Ursula von der Leyen, the European Commission claims to follow the ideal of a “geopolitical Commission” (von der Leyen 2019). It has expanded previous investment schemes that seek to stimulate an expansion of industrial production in the EU. Furthermore, large-scale strategies such as the New Industrial Strategy (COM (2020) 102 final) and Shaping Europe’s Digital Future (COM(2020) 67 final) are aiming for strategic autonomy, particularly in high-tech value chains. Finally, the war in Ukraine is extending the EU’s efforts to achieve further autonomy in the energy sector. As a consequence, fiscal measures are increasingly focussed on that undertaking (Eurogroup 2022).

Overall, against the backdrop of intensified global competition, the state-interventionist strategies of China and other rising powers have also reflected back on the EU’s fiscal and industrial policy. Significant political and private-sector pressure is gathering behind these initiatives, making it unlikely that they will be abandoned (Schneider 2022). However, the new industrial policy may well be made compatible with the austerity paradigm through savings elsewhere (Wigger 2019). The fact that fiscal restraint poses serious limits to the EU’s geoeconomic responses is reflected in the market-based model on which many of its instruments rely: Minor public funds are serving as guarantees for much larger private investments in ventures the member states deem strategically relevant — rather than nation states financing them on their own.

### Structural power relations

The continuities of the inner-European balance of power are particularly informative as to why the RRF has not been followed up by comparable instruments in response to the energy crisis. The austerity-political management of the euro crisis has benefitted strongly from the northern countries’ extended advantages in price competitiveness and industrialization levels (Becker and Jäger 2012). These structural asymmetries have been consolidated rather than mitigated in the last decade, which reflects in the above-mentioned divergences of economic outputs, debt levels, and refinancing conditions. Against this backdrop, the hopes of proponents of fiscal integration rested on two factors in particular: a strengthening of the Italian-French tandem under Draghi and Macron, and a readjustment of German preferences under the social-liberal-green government of Chancellor Olaf Scholz that could shift the entrenched alliances.

Indeed, Macron’s vision of a more fiscally active economic governance found its expression in the RRF to which the German government—to the surprise of many—gave its approval. Former ECB president Draghi, who assumed the office of Italy’s prime minister in February 2021, joined Macron in his demands to perpetuate such mechanisms in the EMU framework (Draghi and Macron 2021). However, by now the Italian-French project appears weakened. Draghi’s government broke down in July 2022. This also drew into question the continuation of the Italian RRF plan—by far the largest in volume. As a consequence, rating agencies downgraded Italy’s credit, worsening its economic outlook (Moody’s 2022). At the same time, Macron’s two-level strategy of reforming the EMU while seeking to thoroughly implement its rules is suffering from strong national resistance (Clegg 2022).

Most importantly, however, Germany’s agreement to the RRF has been misinterpreted to some degree. In the later stages of the euro crisis, chancellor Merkel and finance minister Wolfgang Schäuble had mostly aligned their interests with a reform-averse alliance including the Netherlands, Denmark, Finland, Sweden and some Baltic states. Together they were blocking reform proposals that went beyond already established plans like expanding the ESM and finalizing the banking union. This alliance seemed to show cracks when Germany changed its stance on the recovery fund plans. Merkel’s strategy was initially to rely on ESM loans for investments (Howarth and Schild 2021). Only after some further deterioration of the economic situation did Germany agree to the setup of a temporary investment facility. The shift happened on short notice. The German constitutional court had ruled negatively on the ECB’s PEPP program, making it riskier to rely on central bank action. It is a valid point to make that, no matter the initial intention, such decisions can create unintended consequences and path-dependencies. However, looking at the German rationale, it is quite clear that the decision was founded not in a German opening to a restructuring of the EMU along fiscal union lines, but in “a temporary fix to deal with rapidly increasing functional pressures to act” (Howarth and Schild 2021: 220). This pressure mostly originated from a shift in the German power bloc that sought to offset the global losses in aggregate demand and keep the internal market intact (Ryner 2022; Schneider 2022). We should also take note of the fact that Germany, together with the Netherlands, Austria, Sweden and Denmark, demanded substantial rebates for their approval of the RRF and the EU budget (European Council 2020). Germany’s governing parties were convinced of the one-off nature of the RRF, meaning that they would establish a temporary instrument without setting precedence (Bulmer 2022).

Following these events, Christian Lindner of the liberal party assumed office as finance minister in the cabinet of chancellor Scholz. A self-described “friendly hawk”, Lindner has made clear his aversion to any substantial reform of the fiscal rules — although no red lines had been drawn in the coalition treaty. He insists that the crisis measures “shouldn’t be seen as a precedent or a prelude to reform of the fiscal rules” and that his government “does not support the idea of repeating the joint issuance of debt” (Chazan 2022). Similar views are propagated by the social-democrats under chancellor Olaf Scholz, who was finance minister when the RRF was set up. Jörg Kukies, Scholz’ top economic advisor, has reconfirmed that his government was “very skeptical” of reforms, calling them a “Pandora’s box” that could render the EMU’s framework irrelevant (Rosca and Smith-Meyer 2022).

The interests of the structurally powerful European business associations are generally compatible with that stance. BusinessEurope, the Brussels-based umbrella organization of European industry and employer’s associations, has been lobbying in favor of the RRF and fiscal stimuli (BusinessEurope 2020). It has also expressed its support for a long-term stabilization fund that would mitigate asymmetric shocks in future. Yet, the access to it should be made “fully conditional on Members States implementing structural reforms” (BusinessEurope 2021: 7) *inter alia* in labor markets, pensions, and the public sector — the sectors typically targeted by austerity measures. The organization approves of a flexible application of the SGP that allows for a less abrupt consolidation path and industrial policy measures, but calls for a “proper enforcement” (BusinessEurope 2021: 3).

All things considered, the movement in the German position reflects a one-off concession to demands of fiscal unionists in light of unprecedented economic turmoil and the search for temporary fixes that would keep the EMU from disintegrating. More fundamental reform measures are currently unlikely to find the approval of the northern countries — and the ones taken so far are unlikely to lead to a slippery slope toward fiscal union. This does not mean that the German stance and the austerity project have not lost some of their intransigence, but the general fiscal orientation of the northern member states and their structural influence remain intact.

### Legal-institutional framework

As argued before, the austerity project after 2010 built strongly on the ordoliberal elements of new constitutionalism that had been enshrined in the EMU’s architecture. This mostly concerned the pronounced focus on fiscal consolidation and debt limits, but also the European rules of competitiveness and their preference for economic solutions with limited public intervention. Against the backdrop of these elements, an austerity project that promoted deep cuts to public expenditure and a pushback on governments’ redistributive functions seemed ideologically consistent.

The main difference between the situation in 2010 and today is that, while the discourses surrounding economic policy have become more open to state interventionist practices and fiscal measures (Renda 2021), the EMU’s institutional framework has not. To the contrary, the euro crisis reforms have increased both the complexity and the robustness of EU constitutionalism. Instead of amending EU law, they have mostly been implemented via intergovernmental treaties to allow for a swifter implementation. The Fiscal Compact requires member states to include into their national constitutions a ‘debt brake’, guaranteeing that national budgets are balanced or in surplus, and automatic correction mechanisms. This has been accompanied by stricter expenditure benchmarks and automated correction and sanctioning procedures for the SGP via the Six-Pack and Two-Pack regulations. By now, the export-oriented, fiscally restrictive ideal economy does not just find its fiscal expression in the SGP, but is also reflected in the macroeconomic specifications of the European Semester, which sets stricter limits for current account deficits than surpluses and puts a cap on wage growth.

While a temporary deactivation of the fiscal framework via the general escape clause was feasible in 2020, the EMU’s fiscal framework demands a return to fiscal consolidation at some point. Avoiding this would require a fundamental reform of the EMU along the lines of an alternative fiscal project. However, at least in terms of the scope and depth of European constitutionalist elements, this seems even more unlikely than in 2010. Crisis constitutionalism and the amendments it made to European law, intergovernmental institutions, and national constitutions have made a reversal of the previous institutionalization steps more tedious. The strong opposition the northern countries have displayed against longer-term moves toward fiscal redistribution and expansion hinders a fundamental reform. This leaves institutional tweaks to the SGP’s targets or a more flexible application as pragmatic options (Truger 2020). The recent proposals by the Commission for a reform of the EMU’s fiscal framework (COM(2022) 583 final) reflect precisely this strategy. However, Germany has been hesitant to accept them as a basis for further negotiations. Overall, crisis constitutionalism has further limited the potentials of a fiscal counter-project to austerity as well as the prospects of initiatives for reform.

## Conclusions

Table 1 schematically summarizes the findings of the previous section. It shows that the geoeconomic context under which the EU operates has changed significantly. State apparatuses and private businesses are pushing for industrial policy measures that are meant to safeguard European competitiveness in light of more aggressive trade and investment policies by China and the US. The fragility of global value chains and energy supply contributes to a rethink that shifts the EU’s focus toward strategic autonomy and a partial reshoring. In order to be effective, a coordinated European response requires the channeling of substantial public funds. However, attempts to make such industrial policy measures compatible with fiscal consolidation have led to a predominant reliance on private investments and to compensating cuts elsewhere.

**Table 1:** Schematic comparison of disposition of framework factors towards a fiscal expansive counter-project (+ + high disposition, + disposition, − adverseness, −− high adverseness)

Contextual factors				
Time period	Economic conditions	Geoeconomic competition	Structural relations	Legal-institutional framework
Euro crisis	−	+	− −	−
Current crisis	−	+ +	− −	− −

Economic circumstances within the EU seem unfavorable toward an alternative fiscal project, with many of the symptoms of today’s crisis resembling those of the previous decade. The inflationary environment and the ECB’s change of course will make it a lot harder to find support for joint financing and fiscal expansion. The central question rather is if the northern countries deem a continuation of the austerity project politically feasible. A return to the Maastricht criteria would trigger an unprecedented wave of cuts which would again asymmetrically affect the periphery. Given that the former program countries were unable to consolidate their households since the euro crisis, adjustments of national budgets will be particularly severe there. It is hard to imagine what an austerity-political restructuration of a country like Italy would look like that would bring its debt of around 145 percent of GDP down to SGP limits.

Still, a look at the prevailing power relations and interests has not indicated that the northern countries are willing to deviate from the previous course. In fact, party-political shifts have not translated into substantial shifts in preferences. Germany, after its concessions regarding the RRF, is set to return to a fiscal consolidation path itself and remains adverse to institutional reform on the EU level. Domestic struggles and unfavorable structural positions are weakening Italy and France, the drivers of a fiscal union project. Finally, European capital is expressing its preference for a slowdown of consolidation and industrial policy measures while maintaining its general stance on structural reform and budget neutrality.

The crisis constitutionalism after 2010 has further enshrined the austerity paradigm in the EU’s framework. As a consequence, the success of a fiscal counter-project is contingent on comprehensive institutional reform, which is difficult to realize. This paper’s findings indicate that the RRF, SURE and the issuance of joint EU bonds should not be taken as antecedents of a fiscal union project replacing or challenging austerity. Rather, they are expressions of a process in which the EMU adapts certain elements of a fiscal integrative agenda—a mobilization of joint EU resources and a prioritization of investments over consolidation—to satisfy proponents of a more active fiscal policy, but also because these elements provide necessary fixes to an order that has been inapt to prevent or dampen the current crisis dynamics. In that sense, the measures certainly changed the economic orientation of the EMU—at least temporarily—and pushed the limits of what is politically feasible in European fiscal governance. Yet, at the same time, the basic features of a fiscally restrictive and individualistic European economic order remain intact. History has shown that neoliberalism is quite capable of producing a limited reconciliation with competing interests that still remains faithful to its fundamental logics (Crouch 2009). The RRF and SURE are temporary structures that were deliberately framed by the northern member states as one-off solutions. As the analysis demonstrated, economic, political and institutional framework conditions stand in the way of a perpetuation of their underlying mechanisms. The absence of comparable responses to the current energy crisis and the rather limited amendments proposed by the Commission following its review of the fiscal framework (COM(2022) 583 final) provide further evidence of this.

From a neo-Gramscian perspective, we should therefore speak of passive revolution in light of substantial shifts that are predominantly geoeconomic in nature. The concept alludes simultaneously to the rigidity of the European austerity project, but also to its need for adjustment, which opens opportunities for progressive advances. The findings are generally compatible with Ryner’s (2022) analysis of the European Green Deal and the RRF in which he attests both of them restorative and progressive elements and expresses some skeptical uncertainty about the latter’s realization in light of unresolved contradictions in the German power bloc. It also resonates with Wigger’s (2019) assessment that while geoeconomic shifts contribute to a strengthened investment and industrial policy agenda in the EU, related measures are made compatible with the austerity paradigm.

On a theoretical level, the paper has highlighted the importance of historical-materialist approaches to EU governance. It proposed viewing policies and institutional reform as part of broader political projects that create significant path-dependencies and require favorable circumstances to be overturned. Such a perspective is of particular use for analyses on EU governance where policy change is further hampered by a constitution-like treaty framework. The practical implications of this paper’s findings are that expectations for a transformative impact of the current crisis should be tempered. After the global financial crisis, the EU’s initial response was to stabilize economic conditions with public stimuli. Already in 2010, however, the member states found that “temporary crisis-related sectoral support measures should be phased out as quickly as possible” and be replaced by “an ambitious and credible structural reform agenda” (Eurogroup 2010). This time around, fiscal measures have been much more coordinated and substantial. The political turn toward consolidation also seems less abrupt. Still, the evidence presented in this paper suggests that the turn is imminent. As a resurgence of the austerity project would further deepen the rifts within the EMU and the eurozone’s societies, it would also give traction to counter-projects. Critical scientific inquiry should thus focus on both, the restoration and adaptation processes of the austerity project and the development of credible alternatives.
